# Free and Cued Recall Memory in Parkinson’s Disease Associated with Amnestic Mild Cognitive Impairment

**DOI:** 10.1371/journal.pone.0086233

**Published:** 2014-01-23

**Authors:** Alberto Costa, Marco Monaco, Silvia Zabberoni, Antonella Peppe, Roberta Perri, Lucia Fadda, Francesca Iannarelli, Carlo Caltagirone, Giovanni A. Carlesimo

**Affiliations:** 1 Behavioural and Clinical Neurology Laboratory, I.R.C.C.S. Fondazione S. Lucia, Rome, Italy; 2 Dipartimento di Medicina dei Sistemi, Rome University “Tor Vergata”, Rome, Italy; University of California, San Francisco, United States of America

## Abstract

The hypothesis has been advanced that memory disorders in individuals with Parkinson’s disease (PD) are related to either retrieval or consolidation failure. However, the characteristics of the memory impairments of PD patients with amnestic mild cognitive impairment have not been clarified. This study was aimed at investigating whether memory deficits in PD patients with amnestic mild cognitive impairment (PDaMCI) are due to failure of retrieval or consolidation processes. Sixteen individuals with PDaMCI, 20 with amnestic mild cognitive impairment without PD (aMCINPD), and 20 healthy controls were recruited. Participants were administered the Free and Cued Selective Reminding Test. An index of cueing was computed for each subject to capture the advantage in retrieval of cued compared to free recall. Individuals with PDaMCI performed worse than healthy controls on the free recall (p<0.01) but not the cued recall (p>0.10) task, and they performed better than aMCINPD subjects on both recall measures (p<0.01). The index of cueing of subjects with PD was comparable to that of healthy controls (p>0.10) but it was significantly higher than that of the aMCINPD sample (p<0.01). Moreover, PD patients’ performance on free recall trials was significantly predicted by scores on a test investigating executive functions (i.e., the Modified Card Sorting Test; p = 0.042). Findings of the study document that, in subjects with amnestic mild cognitive impairment associated to PD, episodic memory impairment is related to retrieval rather than to consolidation failure. The same data suggest that, in these individuals, memory deficits might be due to altered frontal-related executive functioning.

## Introduction

Parkinson’s disease (PD) is frequently associated with cognitive impairment. Cross-sectional studies on the prevalence of dementia indicate that it occurs in about 20–40% of patients [1] and longitudinal investigations report prevalence up to 80%. [2] Great efforts have been made to study subtle cognitive deficits that are not associated with functional decline in daily living but could represent the prodromal phase of dementia in PD. Recently, the term Mild Cognitive Impairment (MCI) [3] was applied to this condition also in PD and formal diagnostic criteria were proposed. [4] Indeed, MCI may affect a large percentage of individuals with PD (about 27%); [5] furthermore, with respect to PD patients without cognitive impairments, it is associated with higher risk of dementia. [5].

In a multicenter study, Aarsland et al. [6] reported that a cognitive impairment classifiable as MCI was present in more than 25% of PD participants. A memory impairment occurred in about 13% of participants, visuo-spatial deficits in 11%, and attention/executive disability in 10%. Although these findings indicate that memory deficits affect a high proportion of individuals with PD, the underlying cognitive mechanism is still being debated. In the general PD population, the core hypothesis considers episodic memory disorders in PD as mainly related to retrieval failure. The so-called “retrieval deficit” hypothesis has been corroborated by results of several studies showing that PD patients are less accurate than healthy controls on free recall tasks but may significantly improve their performance on recognition tasks, [7], [8] and by findings that PD patients are significantly facilitated in the retrieval of studied items by the presentation of perceptual or semantic cues. [9], [10] These findings are in line with the idea that cognitive deficits in PD, particularly in the first stages, are mainly related to altered activity of the neural pathways that connect the anterior striatal structures with mesial and dorsal prefrontal regions, which would be affected early by dopamine depletion. [11].

Other behavioural studies report partially divergent data. Davidson et al. [12] and subsequently Cohn et al. [13] showed that PD patients had significant difficulty in performing recognition tasks with respect to controls. Moreover, Higginson et al. [14] reported that PD individuals (both with and without dementia) were significantly impaired with respect to healthy controls in the semantic cued recall of a previously studied word list, and that their degree of impairment on free recall tasks was indistinguishable from that observed on recognition. These findings might support the idea that the memory disorders of PD patients are related to difficulty in consolidating information because of altered activity in dedicated brain areas. Congruently with this hypothesis, a significant association between decreased performance on episodic memory tests and grey matter microstructural (i.e., increased mean diffusivity) [15] and macrostructural (i.e., atrophy) [16] changes in the hippocampal formation of PD patients was reported.

To date, only one study has reported data suggesting that memory deficits in PD patients with MCI may be due to retrieval failure. [17] In fact, results of the study suggest that PD patients with MCI might benefit more from the presentation of a semantic cue in the retrieval phase than individuals with amnestic MCI without PD. Nevertheless, some factors limit the possibility of drawing conclusions from the above. [17] First, the neuropsychological profile of MCI in PD patients was not specified; second, it seems that only immediate (and not delayed) recall was tested; third, the difference between subjects’ free recall and cued recall performance and the facilitation effect of semantic cue presentation were not reported.

The present study was aimed at investigating whether memory deficits in PD patients with amnestic MCI (PDaMCI) are due to a failure of retrieval or of consolidation processes. To investigate this issue, participants were administered the Free and Cued Selective Reminding paradigm [18], which allows controlling deep encoding of the study material and provides effective cues at retrieval. The use of this procedure was suggested by Dubois et al. [19] to discriminate memory disorders due to temporal-mesial related consolidation weakness from deficits due to frontal-related retrieval failure. The performance of PDaMCI (isolated or associated with other cognitive changes) on the above paradigm was compared with that of a group of healthy controls and of individuals with aMCI (isolated or associated with other cognitive changes) which could be attributed to the prodromal Alzheimer’s disease state.[19]–[21] According to the hypothesis that memory disorders are mainly related to retrieval failure in PD, we predicted that PDaMCI patients would perform worse than healthy controls on free but not semantic cued recall. Based on evidence suggesting that memory disorders are due to a hippocampal consolidation failure in individuals with aMCI without PD,[19]–[21] we also predicted that PDaMCI patients would benefit more from cue presentation than individuals with amnestic MCI without PD.

## Materials and Methods

### Subjects

Sixteen PDaMCI individuals, 20 patients with aMCI without PD (aMCINPD), and 20 healthy controls (HC) participated in the study. Sociodemographic and clinical characteristics of the samples are reported in Table 1.

**Table 1:** Socio-demographic and clinical characteristics of the individuals in the three experimental groups.

	Healthy ControlsN = 20	PDaMCI N = 16	aMCINPD N = 20	Fisher F (df = 2,53)	P value
Male/female	11/9	10/6	11/9		
	Mean (SD)				
Age	67.5 (6.0)	66.1 (8.1)	69.7 (6.6)	1.28	>0.20
Education (years)	12.8 (4.2)	11.7 (4.6)	10.9 (4.9)	0.86	>0.40
MMSE	29.1 (1.2)*	27.3 (1.9)	25.9 (2.6)	17.4	<0.001
CDR		0.5	0.5		
UPDRS		25.5 (11.5)			
Hoehn & Yahr scale		2.3 (0.7); = 1 - two subjects; = 2 -six subjects; = 2.5 - one subject; = 3 - seven subjects			
Duration of Disease		4.9 (3.8)			
Apathy evaluation scale		Range: 21–36; Mean = 30.1; SD = 4.5			
Beck Depression Inventory		Range: 3–14; Mean = 8.0; SD = 3.6			
ADL		Range: 4–6	All subjects = 6		
IADL		Range: 5–8	All subjects = 8		
Pill Questionnaire		>2			

* Indicates a significant difference from both aMCIPD and aMCINPD subjects (p<0.01 in both cases) resulting from application of Tukey’s HSD test for unequal samples.

UPDRS: Unified Parkinson’s disease Rating Scale-Part III [34].

PDaMCI: PD patients with amnestic mild cognitive impairment (isolated or associated with other cognitive changes).

aMCINPD: individuals with amnestic mild cognitive impairment (isolated or associated with other cognitive changes) without PD.

#### Ethics statement

The study was conducted in compliance with the principles stated in the Declaration of Helsinki with material and procedures approved by the Ethics Committee of Fondazione S. Lucia. Subjects participated in the study after giving their written informed consent.

#### PDaMCI individuals

Idiopathic PD was defined according to the United Kingdom Parkinson’s Disease Society brain bank criteria. [22] Inclusion criteria included: i) absence of dementia based on Diagnostic and Statistical Manual of Mental Disorders criteria (DSM-IV) [23] and a Mini Mental State Examination score (MMSE) ≥26; [24], [25] *ii*) diagnosis of aMCI according to Litvan et al.’s criteria [26], which includes: a) cognitive complaints corroborated by an assistant; b) pathological performance (i.e., according to normal cut-off scores corresponding to a performance ≥95% of the lower tolerance limit of the normal population distribution, that corresponds to about 2SD from the reference mean) on two neuropsychological tests, at least one of which investigated episodic memory; c) absence of a significant impact of the cognitive disorder on functional daily living as indicated by a score on the Activity and Instrumental Activity of Daily Living [27] and on the Pill questionnaire [25] consistent with minimal changes in routine activities management; d) no evidence of major depression according to the DSM-IV criteria. [23] Accordingly, three patients had single domain aMCI and 13 aMCI multiple domains (pathological performance on tests tapping episodic memory plus executive or visual-spatial dysfunction). It should be noted that according to Litvan et al. [26] amnestic/non-amnestic terminology should not be used to define MCI in PD. Nevertheless, we chose to use this traditional terminology (widely used in the MCI literature) to better highlight the different memory profiles of PD and non-PD patients with MCI.

The Pill questionnaire is administered to both patients and caregivers. This instrument investigates patients’ ability to manage the dopamine treatment suggested by the task force of the Movement Disorder Society to assess the impact of cognitive decline on the activities of daily life; it takes into account the effects of motor disorders [25]. According to Dubois et al. [25], there is no impact of cognitive disorders on daily life if patients are able to describe the drugs, doses and timing of therapy or if they need help from the examiner but the caregiver certifies that they can safely and reliably take the pills without supervision (score = 3). There is an impact on daily living if the caregiver reports that the patient cannot take the pills without supervision or if the patient is unable to describe (even with the help of the examiner) the drugs, doses and timing of the dopamine therapy (scores of 2 and 1, respectively). The Beck Depression Inventory [28], [29] and the Apathy Evaluation Scale – Patient Version [30], [31] were also administered to assess severity of depression and apathy, respectively. At the time of the assessment, PD patients were being treated with levodopa and/or dopamine agonists (pramipexole, ropinirole; rotigotine; levodopa equivalent: mean = 611.8; SD = 216.9). No patient was taking any drugs that affect the central nervous system other than dopamine compounds.

#### aMCINPD

Inclusion criteria included: [32] a) subjective memory complaint corroborated by an assistant; b) pathological score on at least one of the neuropsychological tests assessing episodic memory; c) absence of dementia based on DSM-IV criteria [23] and performance score above 24 on MMSE; [24] d) no or very mild impact of the memory deficit on daily living activities, as indicated by a normal score on the Instrumental Activities of Daily Living scale [27] and by a total score of 0.5 on the Clinical Dementia Rating Scale (CDR) [33]; e) no evidence of any pathology able to induce memory disorders, as indicated by normal thyroid functioning, vitamin B12 and folic acid serum levels, syphilis serologic results, neurological examination, and negative CT or MR brain imaging results for focal lesions (minimal diffuse changes or minimal lacunar lesions of white matter were accepted); f) no evidence of major depression according to DSM-IV criteria. [23] Accordingly, five individuals had single domain aMCI and 15 aMCI multiple domains (pathological performance on tests tapping episodic memory plus executive or visual-spatial dysfunction).

#### Healthy individuals

Inclusion criteria included: *i)* absence of current or previous neurological or psychiatric disorders; *ii)* no history of alcohol or drug abuse; *iii)* absence of subjective memory disturbance; *iv)* MMSE score >24. [24] In order to verify the presence of false negatives in the recruitment of individuals in the HC group, we examined if some of these subjects had outlier scores on the experimental memory procedure. We found that only one subject fell below 2 SD from the group mean on the cued recall. No other outlier scores were found.

### Neuropsychological Test Battery

Standardized tests were administered to both PDaMCI and aMCI individuals without PD to assess episodic memory (Immediate and Delayed Recall of a 15-Word List, [35] Prose Recall, [36] Rey’s Figure [36]), attention and short-term memory (Digit Span and Corsi Block Tapping test Forward and Backward, [37] the Trail Making Test [38]), executive functions (Phonological Word Fluency, [35] Modified Card Sorting test [39], Raven’s Coloured Progressive Matrices [35]), language (Objects and Verbs Naming subtests from the Neuropsychological Examination of Aphasia [40]) visual-spatial functions (Copy of Drawings and Copy of Drawings with Landmarks [35]).

### Free and Cued Selective Reminding Test

#### Material

We used a modified version of the original paradigm [18]. The material consisted of six stimulus tables, each representing four figures of concrete objects. Twenty-four figures belonging to 12 different semantic categories (flowers, musical instruments, animals, desserts, clothing, vegetables, vehicles, jobs, furnishings, drinks, tools and fruits) were presented. The four pictures in each table belonged to four different semantic categories.

#### Procedure

In the study phase, the six tables are individually presented to the subject. The examiner names a category and the subject is required to name and point to the picture that belongs to that category. For instance, when the examiner says “clothes” the subject has to name and point to the item representing a “tie”. After all four items have been identified, they are covered and the subject has to retrieve the studied items that can be classified in the categories named by the examiner. If the subject fails to recall one or more items, the table with the four pictures is shown again and the above procedure is repeated until the subject accurately retrieves all four items. This procedure is repeated for all six tables. Then two test phases are performed. In the first one, after a 20-second delay in which the subject is engaged in an attentional task (i.e., counting backwards from 20 to 1), a free recall test is administered. For the items the subject fails to recall freely, a subsequent cued recall is performed according to the above modalities. This phase (free recall followed by cued recall) is repeated three times in a row.

In the second phase of the test, after a 15-minute delay, during which subjects are administered cognitive tasks that do not involve memory and/or learning (e.g. constructional praxis test), a single free recall test followed by a single category cued recall test are administered. The procedure is the same as that used to test immediate recall. Also in this case, there is no time constraint.

Free and cued recall accuracy is recorded for both immediate and delayed trials. For free recall, the number of recalled items is computed (range: 0–24 for each trial); for cued recall, accuracy is computed by adding to the free recall score the number of items recalled in the following cued recall task (range: 0–24 for each trial). As for immediate recall, for the purpose of statistical analysis only the total score obtained by subjects in the last trial was considered.

Cued recall accuracy computed according to the above modalities is a spurious measure of the effectiveness of semantic cues. In fact, as the cued recall task is performed only on the items that were not recollected in the free recall task, the cued recall score depends greatly on the free recall performance. To control for this effect, we computed an Index of Sensitivity of Cueing (ISC) [41] for both immediate and delayed recall according to the following formula: (free recall score–total recall score)/(free recall score–total items). In the statistical analyses, we used the ISC to quantify the potential facilitation effect of cued compared to free recall.

Subjects were administered the Free and Cued Selective Reminding Test and the neuropsychological tests battery in two different days, with an inter sessions delay of about one week.

### Statistical Analysis

Repeated-measures ANOVAs were applied to accuracy scores on immediate and delayed recall Tasks with Group (PDaMCI vs. aMCINPD vs. HCs) as between-subjects factor and Task (Free vs. Cued recall) and Trial (Immediate vs. Delayed) as within-subjects factors. A similar analysis was applied to ISC with Trial (immediate vs. delayed) as the only within factor. In the case of significant main effects, we performed post hoc analyses with the Tukey HSD test.

Forward stepwise linear regression analyses, with neuropsychological tests scores as explicative factors and free recall scores as dependent variable, was performed to evaluate the predictive value of executive and episodic memory indices on free recall accuracy.

## Results

### Cued Selective Reminding Test

**Figure 1:**
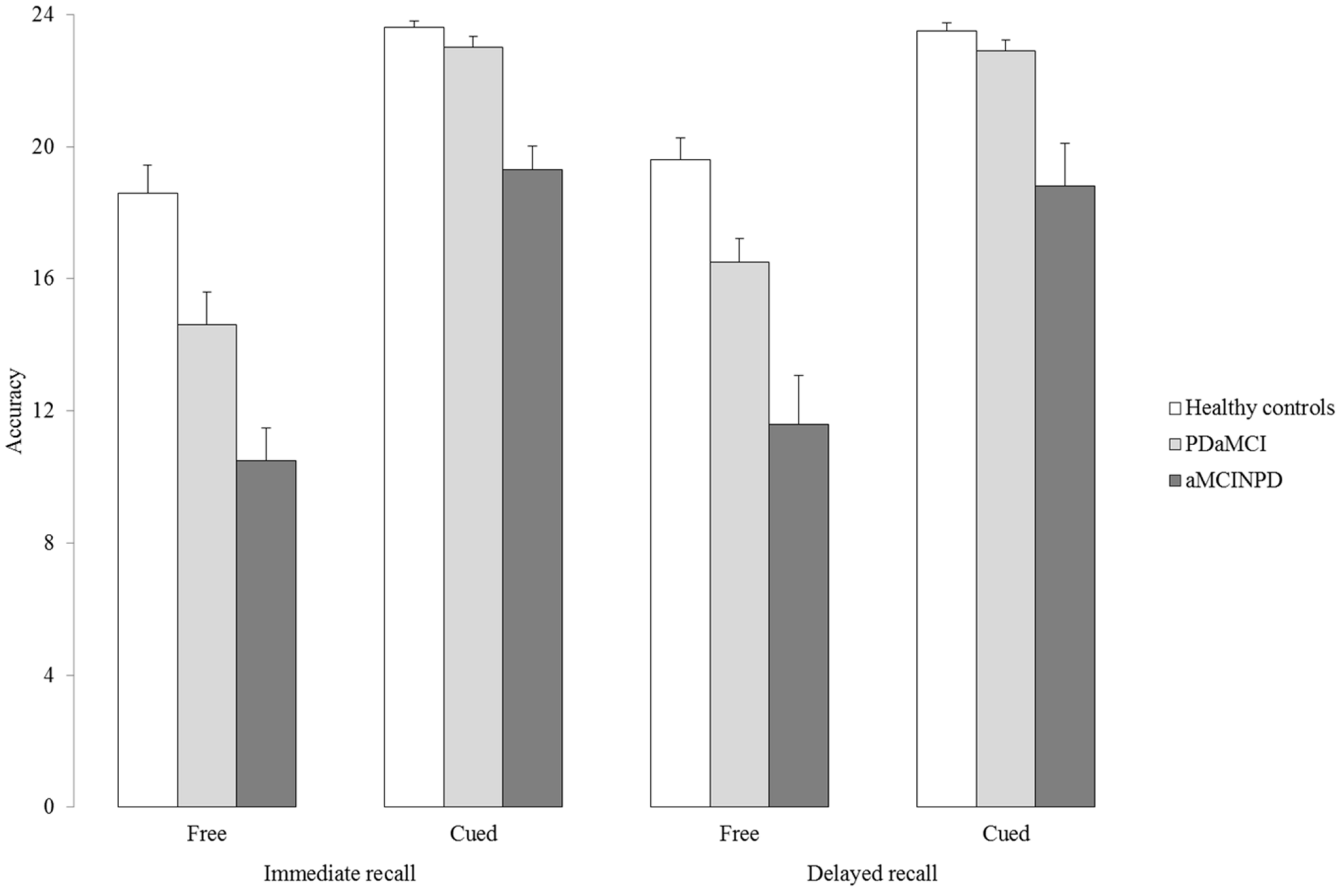
The figure illustrates average accuracy scores achieved by subjects in the three experimental groups on both free and cued recall tasks. Vertical bars represent standard errors.

Figure 1 illustrates subjects’ performance. The main effect of Group (F(2,53) = 20.4; p<0.001) and Task (F(1,53) = 336.6; p<0.001) were significant; the effect of Trial approached statistical significance (F(1,53) = 3.14; p = 0.082). The Group*Task (F(2,53) = 9.85; p<0.001) and Task*Trial (F(1,53) = 8.18; p<0.01) interactions were also significant. Tukey’s HSD test showed that, compared with HC (free recall: mean = 19.1; SD = 3.4; cued recall: mean = 23.5; SD = 1.1), PD patients performed worse on free recall (mean = 15.6; SD = 3.5; p<0.001; Cohen’s *d* [42] = 1.01) but not on cued recall (mean = 22.9; SD = 1.3; p>0.90; Cohen’s *d* = 0.52) of target items, regardless of the trial modality (immediate or delayed); compared with aMCINPD (free recall: mean = 11.1; SD = 5.5; cued recall: mean = 19.1; SD = 4.6) they performed significantly better on both free and cued recall tasks (p<0.001 in all cases; Cohen’s *d* = 1.00 and 1.29, respectively). In turn, the aMCINPD group performed poorer than HCs on both recall measures (p<0.001 in both cases; Cohen’s *d* = 1.18 and 1.58, respectively). Moreover, in the whole experimental sample immediate free recall was significantly lower (mean = 14.6; SD = 5.3) than delayed free recall (mean = 15.9; SD = 5.7; p<0.01); no significant difference was found between immediate (mean = 21.9; SD = 2.8) and delayed cued recall (mean = 21.7; SD = 4.2; p>0.90). Since the performance of one HC subject on the cued recall fell below 2 SD from his group mean, principals analyses were repeated removing this subject. Results confirm the statistical significance of both the Group effect (F(2,52) = 21.9; p<0.001) and of the Group*Task interaction (F(2,52) = 9.69; p<0.001). Post hoc tests also confirm that, in respect to HCs, PD patients performed worse on free recall (p<0.001) but not on cued recall (p>0.80) of target items.

**Figure 2:**
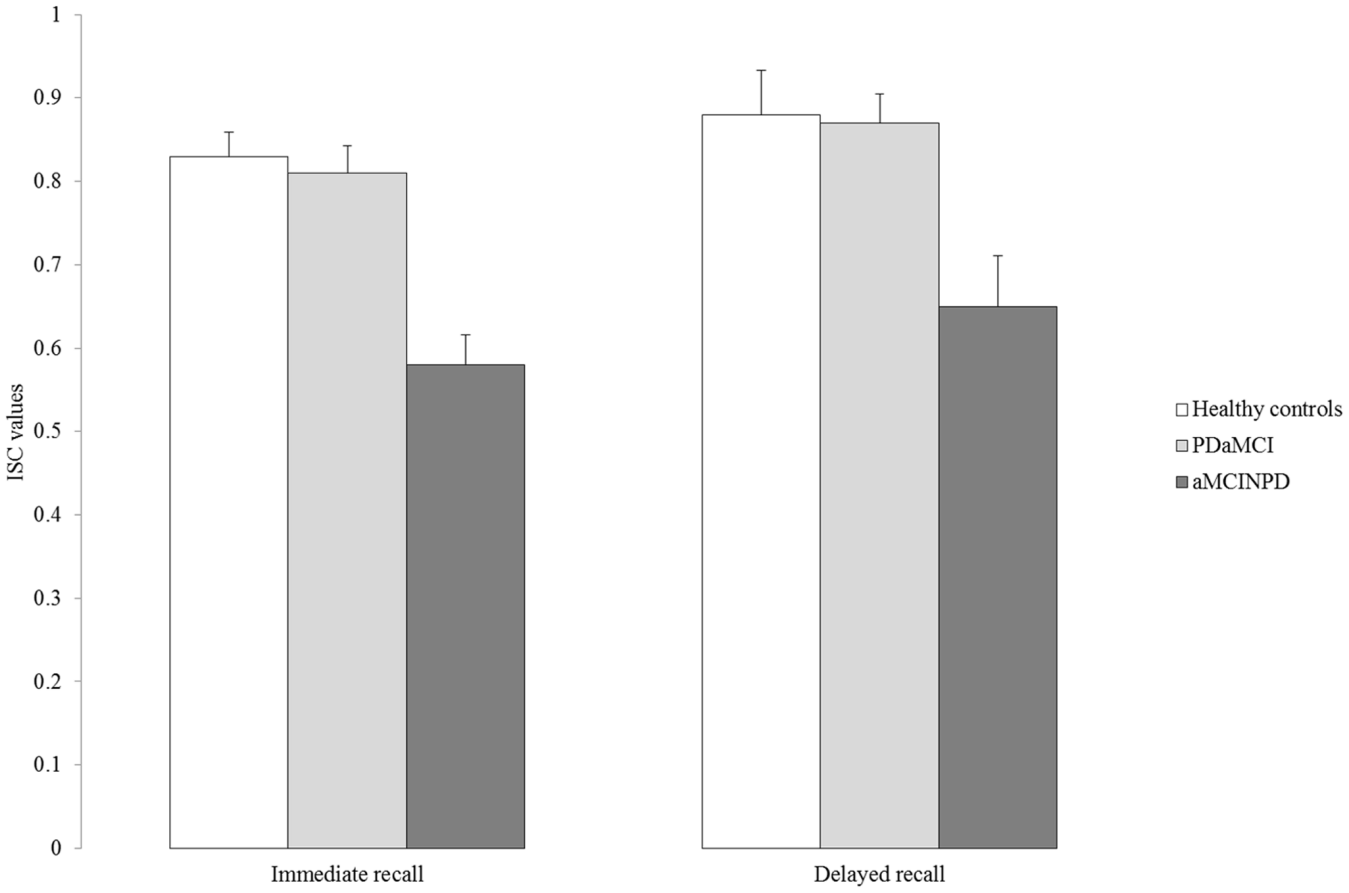
The figure illustrates Index of Sensitivity of Cueing average values showed by subjects in the three experimental groups on immediate and delayed trials. Vertical bars represent standard errors.

Figure 2 illustrates subjects’ ISC values. ANOVA showed that the main effect of Group was significant (F(2,53) = 12.4; p<0.001). Post hoc tests showed that PD patients’ ISC values (mean = 0.84; SD = 0.14) were comparable to those of HC (mean = 0.85; SD = 0.19; p>0.90; Cohen’s *d* = 0.06) but higher than those of aMCINPD (mean = 0.62; SD = 0.22; p = 0.001; Cohen’s *d* = 1.22) individuals. In turn, the aMCINPD group showed lower ISC values than HC (p<0.001; Cohen’s *d* = 1.12). The effect of Trial was also significant (F(1,53) = 5.50; p = 0.023), documenting that the ICF values were higher for delayed (mean = 0.80; SD = 0.25) than immediate recall (mean = 0.74; SD = 0.18). The Group*Trial interaction was not significant (F(2,53) = 0.07; p>0.10). Also in this case, we repeated analyses removing above HC subject. The effect of the Group remained significant (F(2,52) = 13.9; p<0.001). Results of HSD tests confirm that PD patients’ ISC values were comparable to those of HCs (p>0.80).

### Relationship between Free Recall Score and Performance on Executive and Episodic Tests in the Patients’ Samples

For the purpose of these analyses, as the subjects performed similarly on immediate and delayed free recall, the dependent variable was the average score achieved on these two trials. The independent variable in the executive domain was the Modified Card Sorting Test score (i.e., categories achieved and perseverative errors), a measure found to be sensitive to prefrontal-striatal related executive dysfunctions of PD. [43] The other independent variable was the Prose Memory score (i.e., average score between immediate and delayed trials), which is considered sensitive to memory disorders due to altered temporo-mesial brain functioning. [44].

In the PD group, the only explicative variable entering the regression equation was the Modified Card Sorting test-perseverative errors (R^2^ = 0.26; β = −0.51; F(1,14) = 4.98; p = 0.042), with an inverse correlation with the dependent variable. This documents that increasing the number of perseverative errors significantly predicts worse free recall. No other independent variable entered the model (p consistently>0.10). In fact, the correlations between the dependent variable and Modified Card Sorting Test-categories achieved (Pearson’s R = 0.33; β = 0.07) and with Prose Memory (Pearson’s R = 0.06; β = 0.05) were non significant.

The same analysis performed in the aMCINPD sample gave different results. In this case the explicative variable entering the regression equation was Prose Memory score (R^2^ = 0.31; β = 0.55; F(1,17) = 7.48; p = 0.014) with a positive correlation with the dependent variable. No other independent variable entered the model (p consistently >0.10; Modified Card Sorting Test-categories achieved: Pearson’s R = 0.02; β = 0.17; Modified Card Sorting Test-perseverative errors: Pearson’s R = −0.11; β = −0.15).

## Discussion

Episodic memory disorders of individuals with PD are hypothesised to be mainly related to retrieval deficits or, alternatively, to consolidation failure. Here, we set out to investigate this issue in PD patients with aMCI. We administered the Free and Cued Selective Reminding Test [18] to a PD sample with aMCI, healthy controls and to subjects with aMCI without PD. We included the latter group because the episodic memory disorders of these individuals are retained to be primarily due to consolidation deficits resulting from well-documented structural changes in the temporo-mesial brain regions. [19]–[21], [45].

Results indicate that memory disorders in PDaMCI patients are related to retrieval rather than storage failure. The following evidence supports this conclusion. First, PDaMCI patients performed worse than HC in the free recall but not the cued recall condition. Second, this pattern of performance was quite different from that of aMCINPD subjects who, with respect to HC, were impaired on both free and cued recall tasks. Third, the facilitation effect of the semantic cue on PDaMCI subjects’ retrieval (i.e., ISC values) was indistinguishable from that observed in HC and was significantly higher than that of aMCINPD persons.

These results are in line with previous findings which showed that PD patients’ memory performance significantly improved when retrieval was assessed with recognition or cued recall paradigms rather than free recall paradigms. [7], [45] These findings have been interpreted in light of the dysexecutive dysregulation reported early in PD. [46] In fact, PD patients show reduced ability to access stored information (particularly in free recall memory tasks) due to difficulty in spontaneously implementing efficient retrieval strategies and decreased attention resources [7]. Altered activity in the prefrontal-striatal dopamine loops have been reported to be the main etiopathogenic factor accounting for such a deficit. [46] In fact, our data can be interpreted within this framework. We found that performance on a task sensitive to frontal-striatal integrity (i.e., the Modified Card Sorting Test [43] perseverative errors), not performance on an episodic memory test (i.e. Prose Memory), significantly predicted the free recall scores of PDaMCI patients. Conversely, in the aMCINPD sample low free recall scores were predicted by poor performance on the Prose Memory test. Thus, we could hypothesize that the need to recruit prefrontal-related executive/attention abilities have affected PDaMCI patients’ performance in the free recall of otherwise correctly stored items. Instead, the relative reduction of executive demands in the semantic cued recall condition could have facilitated PD patients’ retrieval.

These data are inconsistent with previous behavioural data indicating that PD patients (with respect to HC) were impaired in semantic cued recall [14] and with results of two neuroimaging studies showing a significant relationship between temporal-mesial structural grey matter alteration and PD patients’ performance on declarative memory tasks. [15], [16] One reason for this discrepancy is that, unlike the above-cited studies, we tested memory abilities specifically in PD patients with aMCI.

Limits of the study are represented by the relatively low sample size that do not allow a reliable comparison between individuals with single and multiple domains aMCI, and some differences in recruitment criteria of the two MCI groups (e.g., the exclusion criteria of abnormal vitamin B12 and folic acid serum levels in the MCINPD but not in the MCIPD group). This observation suggests cautions in generalizing our findings to the general PD population. Despite these notes of caution, the results of this study are particularly interesting from a clinical perspective. Indeed, MCI is considered the prodromal phase of dementia. [3], [20] This also seems to be the case in MCI associated with PD [5]. In fact, with respect to PD patients without MCI, individuals with PD and MCI have been found to have a greater risk of developing dementia. [5] However, the profile of the memory disorder may be highly informative for predicting the phenomenological features of dementia. In this regard, Dubois et al. [19], [20] suggested using the memory procedure we adopted here (i.e., the Free and Cued Selective Reminding test) to identify Alzheimer’s disease early. The authors argued that in an individual who performs pathologically on this test, the absence of a semantic cue-related facilitation effect on retrieval would reveal difficulty in consolidating information, indicating dysregulated hippocampal activity, as precociously observed in Alzheimer’s dementia. [47] In PD, dementia may present with both neuropsychological and neurobiological signs typical of subcortical syndromes and, in a lower proportion of patients, with features resembling those observed in individuals with Alzheimer’s type dementia. [48] Congruently with previous data obtained from PD patients with MCI [17], our results indicate that the free and cued recall paradigm may help discriminate between memory disorders in PD individuals with aMCI and those observed in individuals with aMCI without PD in which the relatively lower sensitivity to the cueing likely indicates a reduced efficiency of consolidation mechanisms.[19]–[21] Therefore, using this paradigm with PD patients might help identify the different forms of cognitive impairment early and allow implementation of the best therapeutic approaches. For instance, in an individual who selectively fails to retrieve in the free recall condition, alongside to a possible pharmacological treatment, cognitive intervention could be planned that is focussed on the improvement of mechanisms of executive control; a different compensatory approach could be adopted with an individual whose memory deficit persists in the cued recall condition. Longitudinal studies should be performed to verify the sensitivity of above paradigm in predicting dementia in PDaMCI patients.
